# New Appliances and Things Medical

**Published:** 1905-06-10

**Authors:** 


### NEW APPLIANCES AND THINGS MEDICAL.

[We shall be glad to receive, at our Office, 28 & 29 Southampton Street, Strand, London, W.C., from the manufacturers, specimens of all new preparations and appliances which may be brought out from time to time.]

### CONULES EPHEDRIN CO. (SETTERIE).—DIASTASE (SETTERIE).

(The British Pharmacal Co., 12 Furnival Street, London.)

The conules before us are a special preparation of the active principle of the suprarenal gland, combined with an astringent substance, called æsculin, derived from the bark of the horse-chestnut. They have approximately the same weight as an ordinary suppository, also a similar shape but are somewhat longer. The conules are essentially prepared for use in cases of hæmorrhoids, and hence can be used systematically, and also on occasion should there be any hæmorrhage after or upon defecation. There is no doubt whatever that they are powerfully hæmostatic, and that by their use the adrenalin can be brought into contact with the injected or bleeding mucous membrane.

*Diastase.* (Setterie).—This is another preparation which is made by the same company. It is a concentrated and pleasant preparation of diastase or the active enzyme of malting barley. It can be used either alone to aid the digestion of amylaceous foods or for the purpose of predigesting such foods for invalids and children. It is claimed for this preparation that its diastatic power is some 10 times as great as that of any malt extract on the market. A book of receipts is given with the preparation, containing directions for its use.

### OPHTHALMIC CAPSULES (DUNCAN).

(Duncan, Flockhart and Co., London and Edinburgh.)

Messrs. Duncan, Flockhart and Co. have had put up in a specially convenient form for ophthalmic practice atropine, cocaine, and yellow oxide of mercury ointments. The ointments are made with the finest neutral white paraffin jelly, and are enclosed in a curiously-shaped gelatin capsule. This capsule is balloon-shaped, with a very elongated neck. The tip of this elongated neck is cut off and the remaining tube applied to whatever part of the eye may be desired. Upon pressure being then made upon the body of the capsule the ointment exudes and can be thus applied to the desired spot. We cannot imagine anything more convenient for pplication to the eye, and feel sure that ophthalmic practitioners will speedily avail themselves of so desirable an addition to their equipment.

